# Prolapsus and Amputation of the Rectum

**Published:** 1890-08

**Authors:** James A. Waugh

**Affiliations:** Veterinarian, Sixth Cavalry, U. S. A.


### PROLAPSUS AND AMPUTATION OF RECTUM.

By JAMES A. WAUGH,
*Veterinarian, Sixth Cavalry, U. S. A.*

A GREY horse mule, aged 12 years, 15 hands high, and used as a troop pack animal, suffered from intestinal obstruction and abdominal pains. A purgative, combined with anodynes and stimulants, was administered early in the morning; clysters of warm soap water were injected into the rectum and the latter was carefully explored with the hand. Rectal injections were repeated every three hours during the day and anodynes were used freely to alleviate suffering. I was called early next morning and found the patient had been exerting himself considerably in endeavoring to defecate, and had caused an eversion of about eight inches of the rectum. The farrier had formerly been a student in Cornell University, and was well versed in Prof. Law's lectures; therefore he concluded to attempt reduction of the everted bowel, and not trouble me until next morning, but he accidentally tore the bowel considerably and then deemed it best to call me. The bowel was greatly swollen and badly lacerated. It was midwinter in the Rocky Mountains, and the patient was confined with other animals in a new frame stable which was rather cold. Therefore, I decided to defer surgical interference till next morning. The everted bowel was washed with warm water and dressed with carbolized oil. A twitch was sufficient to restrain the animal. Interrupted silk sutures were introduced through the anus and bowel superiorly, laterally and inferiorly, then secured so as to include about an inch of the tissue. A tenaculum and scalpel were used to remove the everted bowel. There was considerable hemorrhage which was soon checked with iced water and astringents. Injections of carbolized oil were used freely to relax the bowels and keep the wounds in good condition. Anodynes were used both day and night to relieve urgent symptoms and alleviate sufferings. The patient improved gradually and made a competent recovery, and is now working in a team. I afterward learned that this pack mule had suffered a similar attack of this trouble while being used on a hunting trip during the holidays, and the farrier had treated it successfully, but never reported the matter to me.

